# Spatial and temporal variations of faecal indicator bacteria in Lake Bunyonyi, South-Western Uganda

**DOI:** 10.1007/s42452-021-04684-4

**Published:** 2021-06-10

**Authors:** Alex Saturday, Thomas J. Lyimo, John Machiwa, Siajali Pamba

**Affiliations:** 1 Department of Molecular Biology and Biotechnology, University of Dar Es Salaam, P.O. Box 35064, Dar es Salaam, Tanzania; 2 Department of Environmental Sciences, Kabale University, P.O. Box 317, Kabale, Uganda; 3 Department of Aquatic Sciences and Fisheries, University of Dar Es Salaam, P.O. Box 35064, Dar es Salaam, Tanzania

**Keywords:** Faecal bacteria, Indicator bacteria, *E. coli*, Enterococci, Lake Bunyonyi, Uganda

## Abstract

*Background* Microbial water quality serves to indicate health risks associated with the consumption of contaminated water. Nevertheless, little is known about the microbiological characteristics of water in Lake Bunyonyi. This study was therefore undertaken to examine the spatial and temporal variations of faecal indicator bacteria (FIB) in relation to physicochemical parameters in Lake Bunyonyi. *Result* The FIB concentration was consistently measured during sampling months and correlated with each other showing the presumed human faecal pollution in the lake. The highest concentration values for *E. coli* (64.7 ± 47.3 CFU/100 mL) and enterococci (24.6 ± 32.4 CFU/100 mL were obtained in the station close to the Mugyera trading centre. On a temporal basis, the maximum values were recorded during the rainy season in October 2019 (70.7 ± 56.5 CFU/100 mL for *E. coli* and 38.44 ± 31.8 CFU/100 mL for enterococci. FIB did not differ significantly among the study stations (*p* > 0.05) but showed significant temporal variations among the months (*p* < 0.05) with concentrations being significantly high in wet season than dry season (*U* = 794, *p* < 0.0001 for *E. coli*; *U* = 993.5, *p* = 0.008 for enterococci). Spearman’s rank correlation revealed that FIB concentrations were significantly positively correlated with turbidity and DO concentration levels (*p* < 0.05). Approximately 97.2% of the water samples had *E. coli* and enterococci concentrations levels below USEPA threshold for recreational waters. Likewise, 98.1 and 90.7% of samples recorded *E. coli* and enterococci counts exceeding the UNBS, APHA, WHO and EU threshold values for drinking water. *Conclusion* The FIB counts show that the Lake Bunyonyi water is bacteriologically unsuitable for drinking unless it is treated since the FIB pose health risks to consumers. Besides, the water can be used for recreational purposes.

## Background

The microbiological quality of freshwater bodies serves to indicate health risks associated with the contamination of water by faecal sources [26]. Contamination of freshwater lakes can result from the influx of runoff from agricultural land after manure applications, faulty septic systems, and direct deposition from people, animals and wastewater discharge. The use of contaminated water for drinking, irrigation and recreation poses health risks to people. Globally, approximately 600 million people suffer annually from waterborne diseases after drinking contaminated water [14]. Consumption of safe and clean water has been increasingly viewed as the best alternative for increasing human safety against waterborne diseases. The bacteriological quality of water in the lake system is recognized as a major factor that affects surface water quality. Therefore, a lake whose water is supplied for drinking, recreation and livelihoods must be assessed to avoid threats from enteric pathogens [20].

Testing for every enteric pathogen is time-consuming and expensive, so regulatory agencies have focused on the enumeration of faecal indicator bacteria (FIB) to assess the bacteriological quality of freshwater sources [31]. Thus, understanding the source of faecal pollution in the lake system is vital as contamination from diverse sources poses varying risk levels. Human sewage and livestock faecal matter are the most high-risk sources of faecal contamination which may contain human-specific viruses and pathogens [17, 22].

The *E. coli* and enterococci bacteria are commonly used indicator microorganisms for bacteriological quality of water intended for various purposes. They are generally non-pathogenic and inhabit the gastrointestinal tract of warm- and cold-blooded animals. They are shed in faeces along with enteric pathogens. Thus, their measure shows the degree of faecal contamination. According to USEPA [31], the safety threshold for *E. coli* concentration in fresh recreational waters was established as a geometric mean of 235 CFU/100 mL in single samples and 126 CFU/100 mL in multiple samples. For enterococci, the safety threshold is at a geometric mean of 104 CFU/100 mL in single samples and 33 CFU/100 mL in multiple samples in a 30-day interval. The World Health Organization (WHO) guidelines for drinking water state that water intended to be used for drinking should be free from *E.coli* and enterococci [32].

Lake Bunyonyi is a major source of water, employment opportunities and a major tourist destination site in Western Uganda. Its watershed is a densely populated area with extensive subsistence farming. The major crops grown are: sweet potatoes, irish potatoes, beans, sorghum, cabbages [21], and barley was recently introduced in the northern part of the lake watershed. Besides, small scale fishing and aquaculture are also carried out in the lake water [18]. Thus, the livelihood activities of the people in the lake watershed largely depend on the health of the lake ecosystem. Lake Bunyonyi watershed is predominantly occupied by rural people whose pit latrines are constructed less than 20 m from the lake. Nevertheless, no study has been conducted to assess the bacteriological characteristics of water in the lake ecosystem, well knowing that the observed eutrophication induced by anthropogenic activities may jeopardize the ecological services this lake renders. Therefore, this study is the first of its kind regarding the current topic of research on Lake Bunyonyi. The main objective of this study was to examine the spatio-temporal variations in *E. coli* and enterococci concentrations in Lake Bunyonyi to provide a basis upon which necessary measures may be taken to preserve its ecosystem health.

## Materials and methods

### Study area and sampling

**Fig. 1:**
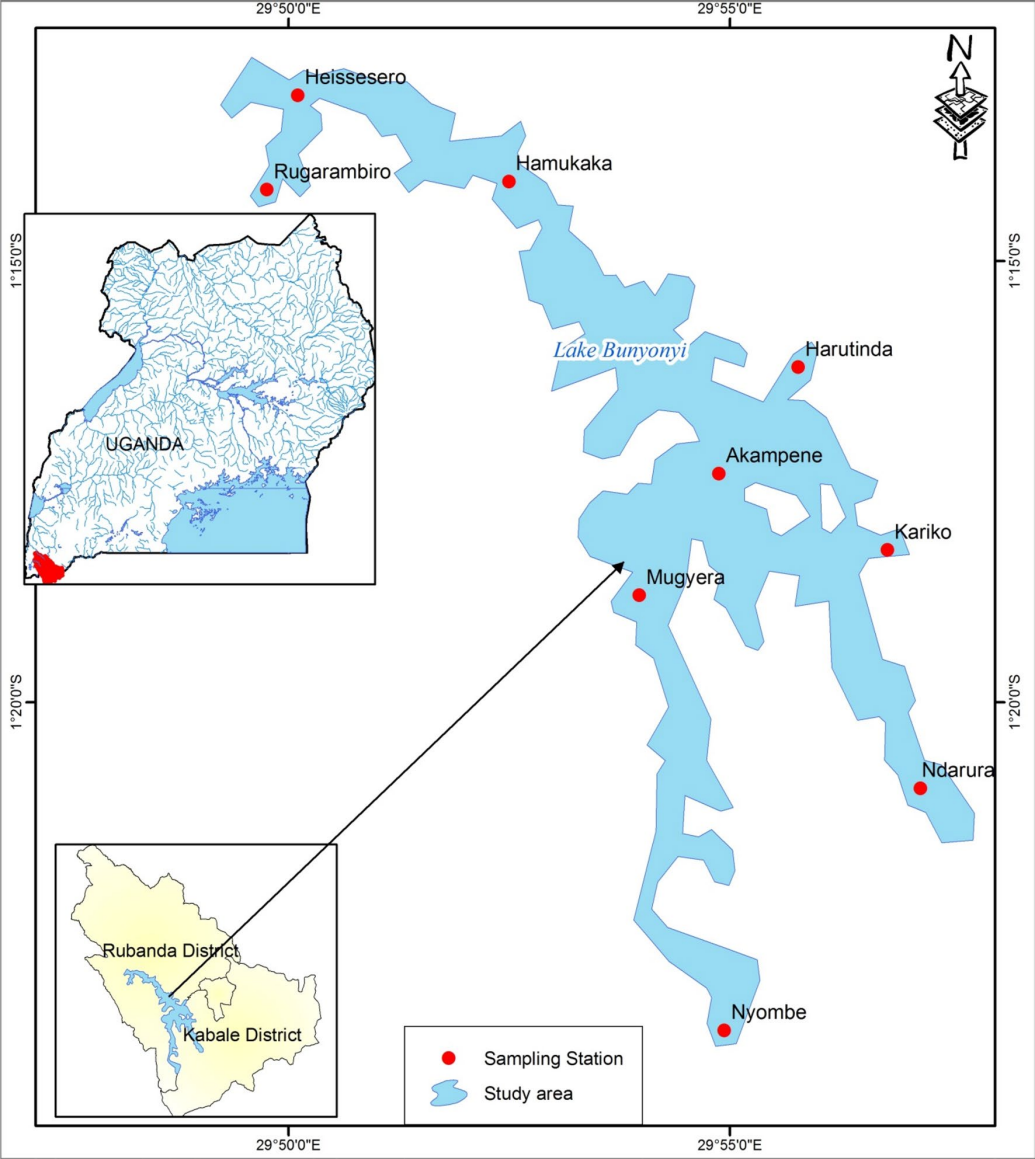
Location Map of Lake Bunyonyi, Southwestern Uganda

The study was conducted on Lake Bunyonyi which is shared by the districts of Kabale and Rubanda in Southwestern Uganda (Fig. 1). Geographically, Lake Bunyonyi is located between 1.2953°S and 29.9133°E and at an average altitude of 1973 m above sea level. The lake is long and narrow with a total surface area of 56 km^2^ with a maximum depth of 40 m [18]. The climate of the study area is warm and temperate influenced by altitude and latitude. It is characterized by a bimodal rainfall distribution with the long rainy season occurring between March and May. The short rains occur between October and November while June to August is the driest period. The mean annual rainfall ranges from 800 to 1000 mm. The temperature in the lake catchment ranges from 23.7 °C in March to 24.8 °C in August [27].

Water samples were collected into 800 mL sterilized glass bottles with corks shielded with aluminium foil for the avoidance of any form of hand contamination. In total, 108 samples were collected from the nine study stations for 12 months (October 2019 – September 2020) for the determination of *E. coli* and enterococci concentrations. All the samples were labelled according to their respective site code (U1–U3, M1–M3 and L1–L3 for samples collected from stations located in the upper, middle and lower Bunyonyi sites (Table 1), respectively to get an overview of the entire lake understudy. All the samples were collected and stored in an icebox with an ice block and transported to the National Water and Sewerage Corporation (NWSC) central laboratories in Kampala for analysis within 24 h. While in the laboratory, samples were stored at 4 °C in the refrigerator waiting for analysis.

**Table 1:** Sampling site location for *E. coli* and Enterococci parameters

Study site	Station code	Station name	Location	
			latitudes	Longitudes
Upper Bunyonyi	U1	Nyombe	1°23′50.16″ S	29°55′08.80″ E
	U2	Ndarura	1°20′58.55″ S	29°57′25.47″ E
	U3	Kariko	1°18′16.30″ S	29°56′46.96″ E
Middle Bunyonyi	M1	Harutinda	1°16′14.98″ S	29°56′16.48″ E
	M2	Akampene	1°17′37.60″ S	29°55′02.47″ E
	M3	Mugyera	1°19′05.62″ S	29°54′09.76″ E
Lower Bunyonyi	L1	Heissesero	1°13′00.32”S	29°49′53.56″ E
	L2	Rugarambiro	1°14′20.11″ S	29°50′04.32″ E
	L3	Hamukaka	1°14′05.76″ S	29°52′29.77″ E

## Analytical methods

### Physicochemical analysis

The physicochemical water quality parameters such as surface water temperature, DO, turbidity, and electrical conductivity (EC) were measured on-site. DO was measured using the DO meter (DO 5510 M.R.C model) while water temperature was measured using a temperature sensor in the DO meter. The pH and EC were measured using a water-resistant hand-held pH meter (HI8314 HANNA instruments) and using a conductivity meter (HI 9033 HANNA instruments), respectively and the turbidity measured was using a turbidity meter (2100P, Hach). All measurements were done in triplicate and the average values are reported herein.

To measure NO_3_-N concentration in water samples, the content of one pillow Nitraver 6 was added to 25 ml of water sample in a graduated mixing cylinder. The mixture was covered with palm, held firmly and inverted several times to dissolve the powder. Between 10 min and 2 h afterwards, absorbance was measured at 543 nm at UV spectrophotometer (DR6000, Hach Laboratory Instruments). For SRP determination in the water sample, 50 mL of filtered water sample was mixed with ammonium molybdate to form molybdo-phosphoric acid in a dry 125 mL Erlenmeyer flask. The acid was reduced by ascorbic acid to a blue complex (molybdenum blue). Thereafter, the colour intensity proportional to the concentration of phosphate in the sample was measured by the spectrophotometer at a wavelength of 880 nm [3]. The NO_3_-N and SRP concentration values were recorded directly from DR 6000 Spectrophotometer in the laboratory.

### Bacteriological analysis

The membrane filtration technique was used for the determination of FIB (*E. coli* and enterococci) concentrations in water samples following APHA [3] standards. Determination of *E. coli* was done by filtration of 100 mL of water samples through a 0.45 μm millipore filter membrane. After filtration, the filters were placed on Chromogenic Coliform Agar (Oxoid Ltd, UK) prepared following manufacturer instructions and then incubated at 44.5 °C for 18–24 h. *E. coil* numbers per 100 ml of a sample were computed as per APHA [3].

For the case of enterococci, 100 mL of water sample was also filtered in the same way as above. However, the membrane filter was placed on Chromogenic agar media (Oxoid Ltd, UK) for enterococci growth and incubated at 41 °C ± 0.5 °C for 48 ± 3 h. Thereafter, the membrane filters were then transferred to a differential medium Esculin Iron Agar (EIA) and then incubated at 41 °C ± 0.5 °C for 30 min. The enterococci colonies on the Me-EIA agar membrane which appeared pink to red on the filter membrane paper developed into black or reddish-brown precipitate on transfer to EIA on the underside of the filter to verify enterococci. All the bacterial count per 100 ml of a water sample analyzed was computed following the standard procedures for microbial analysis by APHA [3].

### Statistical analysis of data

The collected data were analyzed using Statistica version 10 [7]. The Kruskal–Wallis test was conducted to determine differences between study sites, within sampling months. The differences between sites addressed spatial variations while differences within sampling months addressed temporal variations. Mann–Whitney U test was used to determine whether there significant differences in FIB concentrations between the dry season and rainy season. Spearman rank correlation was performed to establish whether there exists a relationship between FIB concentrations and physicochemical parameters. All statistical tests were considered significant at a confidence level of 95% (*p* < 0.05).

## Results

### Physicochemical characterization of study sites

**Fig. 2:**
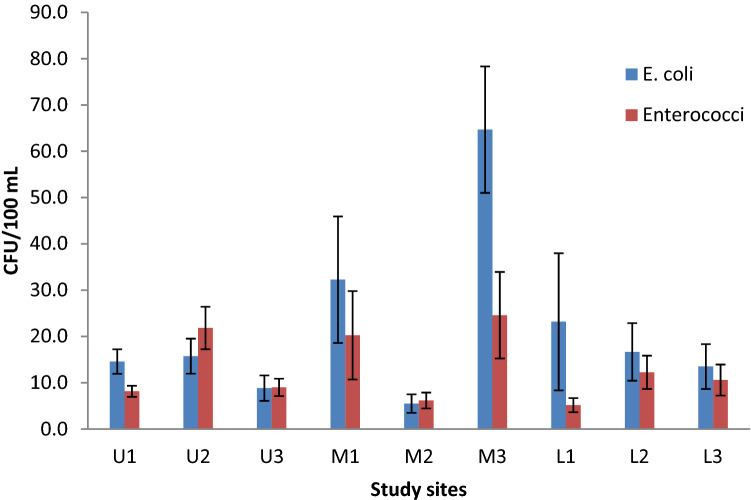
Mean FIB counts across the different study stations during the study period (October 2019–September 2020)

The mean values of the physical and nutrient parameters in the study stations are summarized in Table 2. The water temperature, DO, pH, turbidity, EC and SRP varied from 20.9 ± 1.1 to 21.7 ± 1.5 °C, 6.6 ± 1.2–7.2 ± 1.7 mg/L, 7.4 ± 0.5–7.9 ± 0.6, 2.8 ± 0.6–4.3 ± 1.6 NTU, 241.75 ± 11.5–266.8 ± 61.9 µS/cm, and 0.05 ± 0.02–0.18 ± 0.26 mg/L, respectively (Table 2). The mean NO_3_-N values were uniformly distributed across sampling stations (0.01 ± 0.01 mg/L). Statistically, no significant differences in the mean water temperature, DO, EC, water turbidity, pH, NO_3_-N, and SRP between study stations were observed (Fig. 2) (*p* > 0.05).

**Table 2:** Physicochemical parameters variation at different sampling stations. Mean values ± standard deviation obtained from different months (n = 12)

Station	Temp (^o^C)	DO (mg/L)	EC (µS/cm)	Turb (NTU)	pH	NO_3_-N (mg/L)	SRP (mg/L
Nyombe (U1)	20.9 ± 1.1	6.7 ± 1.4	241.8 ± 11.5	3.1 ± 1.2	7.5 ± 0.5	0.01 ± 0.01	0.18 ± 0.26
Ndarura (U2)	21.7 ± 1.4	6.8 ± 1.4	243.3 ± 7.0	2.8 ± 0.6	7.7 ± 0.5	0.01 ± 0.01	0.11 ± 0.20
Kariko (U3)	21.7 ± 1.5	7.0 ± 1.3	247.7 ± 12.3	3.2 ± 1.2	7.8 ± 0.4	0.01 ± 0.01	0.05 ± 0.02
Harutinda (M1)	21.3 ± 1.5	7.2 ± 1.7	266.8 ± 61.9	3.8 ± 1.7	7.7 ± 0.4	0.01 ± 0.01	0.05 ± 0.02
Akampene (M2)	21.3 ± 1.5	6.8 ± 1.0	243.7 ± 8.1	2.8 ± 0.6	7.9 ± 0.6	0.01 ± 0.01	0.09 ± 0.13
Mugyera (M3	21.3 ± 1.6	6.5 ± 1.2	241.1 ± 11.1	4.3 ± 1.6	7.4 ± 0.5	0.01 ± 0.01	0.05 ± 0.02
Heissesero (L1)	21.0 ± 1.6	6.9 ± 1.3	245.2 ± 8.6	2.9 ± 0.8	7.8 ± 0.7	0.01 ± 0.01	0.05 ± 0.03
Rugarambiro (L2)	21.2 ± 1.4	7.0 ± 1.3	245.8 ± 7.3	3.2 ± 0.8	7.7 ± 0.5	0.01 ± 0.01	0.14 ± 0.33
Hamukaka (L3)	20.9 ± 1.5	6.6 ± 1.2	244.0 ± 7.6	3.6 ± 1.2	7.7 ± 0.7	0.01 ± 0.01	0.05 ± 0.02

### Spatial variation in *E. coli* and enterococci concentration

**Fig. 3:**
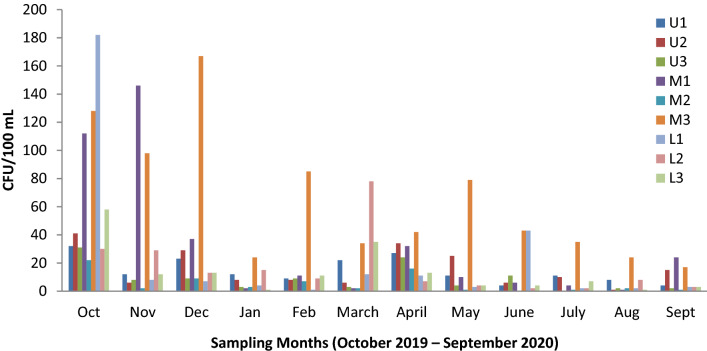
Monthly variation of *E. coli* counts across stations during the study period (October 2019–September 2020)

**Fig. 4:**
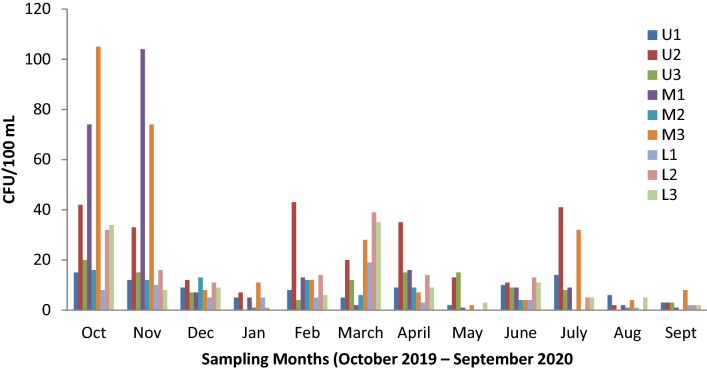
Monthly variation of Enterococci counts across stations during the study period (October 2019–September 2020)

Across the stations, FIB counts ranged from 0 to 182 CFU/100 mL for *E. coli* (Fig. 3) and 0 to 105 CFU/100 mL for enterococci (Fig. 4). The highest mean value for *E. coli* (64.7 ± 47.3 CFU/100 mL) and enterococci (24.6 ± 32.4 CFU/100 mL) were obtained at Mugyera (M3) station (Fig. 2). The least concentration values for *E. coli* (5.5 ± 7 CFU/100 mL) and enterococci (5.2 ± 5.3 CFU/100 mL) were obtained at Akampene (M2) and Heissesero (L1) stations, respectively (Fig. 2). Indeed, Kruskal–Wallis test revealed significant differences in *E. coli* concentration values across stations (*H* (8, N = 108) = 32.004, *p* = 0.0001). Enterococci concentration were not significantly different across study stations (*H* (8, *N* = 108) = 13.8, *p* = 0.087).

### Temporal variation of *E. coli* and enterococci concentration

**Fig. 5:**
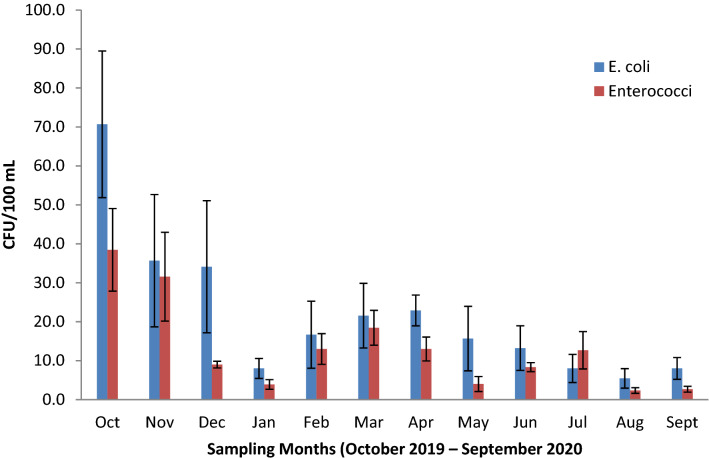
Mean FIB concentration values in Lake Bunyonyi during the study period (October 2019–September 2020)

The results indicate the heterogeneous distribution of faecal contamination between seasons for both *E.coli* and enterococci during the study period. The highest level of FIB count was recorded in October 2019 with a mean value of 70.7 ± 56.5 CFU/mL for *E. coli* and 38.4 ± 31.8 CFU/100 mL for enterococci. On the other hand, the least FIB concentration was obtained in August 2020 with a mean value of 5.4 ± 7.5 CFU/ 100 mL for *E. coli* and 2.3 ± 2.2 CFU/ 100 mL for enterococci bacteria (Fig. 5). Statistically, Kruskal–Wallis test revealed significant differences in FIB counts between study months (*H* (11, *N* = 108) = 37.4, *p* < 0.001 for *E. coli*; *H* (11, N = 108) = 56.6, *p* < 0.001 for enterococci). Considering wet and dry seasons, the mean FIB concentration of the wet season (29.8 ± 40.4 CFU/100 Ml for *E. coli*; 16.7 ± 22.0 CFU/100 mL) were significantly higher than the values (10.3 ± 15.3 CFU/100 mL for *E. coli*; 8.0 ± 9.4 CFU/100 mL) of the dry season (Table 3). Indeed, the Mann–Whitney U test revealed significant differences in FIB concentration between seasons (*U* = 794, *p* < 0.001 for *E. coli*; *U* = 993.5, *p* = 0.008 for enterococci).

**Table 3:** Seasonal variation of FIB in Lake Bunyonyi (October 2019–September 2020)

Season	*E. coli*				Enterococci			
	Means	Std. Dev.	Min	Max	Means	Std. Dev.	Min	Max
Wet season	29.8	40.4	1.0	182.0	16.7	22.0	0.0	105.0
Dry season	10.3	15.3	0.0	85.00	8.00	9.40	0.0	43.00
All Grps	21.7	33.7	0.0	182.0	13.1	18.3	0.0	105.0

The mean FIB values were significantly different between seasons (*p* < 0.05)

The Spearman’s rank correlation was computed to establish whether there were significant relationship between the measured FIB concentration and the physicochemical parameters. The correlation results (Table 4) indicated that *E. coli* was significantly positively correlated with DO (*rs* = 0.390, *p* < 0.05) and turbidity (*rs* = 0.438, *p* < 0.05) but significantly negatively correlated with pH (*rs* = -0.201, *p* < 0.05). Likewise, enterococci was significantly positively correlated with DO (*rs* = 0.365, *p* =  < 0.05) and turbidity *(rs* = 0.211, *p* < 0.05) (Table 4).

**Table 4:** Correlation analysis for FIB and environmental parameters; Values with an asterisk are significant at *p* < 0.05

Variables	E. *coli* (CFU/100 mL)	Enterococci (CFU/100 mL)	Temp (^o^C)	DO (mg/L)	EC (µS/cm)	Turb (NTU)	pH	NO_3_-N (mg/L)	SRP (mg/L)
*E. coli*	1								
Enterococci		1							
Temp	− 0.134	− 0.056	1						
DO	0.390*	0.365*	0.194*	1					
EC	0.065	0.125	0.579	0.361*	1				
Turbidity	0.438*	0.211	− 0.033	0.471*	0.164	1			
pH	− 0.201*	− 0.153	0.153	0.402*	− 0.001	0.108	1		
NO_3_-N	− 0.068	0.082	− 0.197*	0.048	− 0.079	0.300*	0.194*	1	
SRP	− 0.144	− 0.047	0.300*	0.146	0.178	− 0.135	0.138	− 0.327*	1

pH = Water pH, *EC* Electric conductivity, *DO* Dissolved Oxygen, Temp = surface water temperature, *NO*_*3*_*-N* Nitrate –Nitrogen, *SRP *Soluble Reactive Phosphorus

## Discussion

### Physicochemical characteristics of Lake Bunyonyi

The results of physicochemical conditions of Lake Bunyonyi showed insignificant differences among stations but varied with seasons and the values were within the range of previous studies on lakes in Uganda and other countries which share similar characteristics [27, 28]. Surface water temperatures did vary significantly between stations perhaps due to the differences in the study locations and slight differences in the sampling time. The significant temporal variations are attributed to the dynamics in seasonal weather patterns within the Lake catchment area. Besides, the Lake understudy is situated between steep hills associated with several barriers that limit solar radiation on the surface waters of the Lake that would otherwise increase the water temperature. The recorded water temperature range corresponded with that reported in the previous study by Tibihika et al. [27] in Lake Bunyonyi and other minor lakes of the Kigezi sub-region. The dissolved oxygen (DO) levels were relatively low which is attributed to a higher rate of decomposition, especially in the wet season. Nevertheless, the observed DO range do not cause stress to aquatic organisms and can rarely cause mortality and reduction of the sensitive species [16]. Similar to our study results, DO levels of 5.7 and 5.7 mg/L have been reported by Tiémoko et al. [28] at Lake Taabo and Kossou, respectively. Likewise, DO levels across the study stations corroborated with those of Hughes [11] in Lake Bunyonyi.

The recorded EC fell below the WHO maximum permissible limits for freshwater bodies (< 1500 μS/cm). These results indicate out rightly that water in the Lake understudy is not considerably ionized and has a relatively low ionic concentration level. Similar to study results, Edoreh et al. [9] reported slightly lower values across sampling stations in the Ugbevwe pond than the observed values in the present study. Turbidity levels were high in the wet season than in the dry season. These values were slightly higher than those reported by Anjusha et al. [2] with a mean turbidity range of 2.5–3.9 NTU in the Periyar River. The high turbidity level may indicate continuous terrestrial influence from rain runoff which carries faecal material and other debris into the Lake ecosystem. Besides, the recorded turbidity level could be attributed to cultivation coupled with infrastructural development on the Lakeshores; all of which accelerate soil erosion and sedimentation, and the resulting suspended solids are deposited into the lake system through surface runoff. The turbidity range values fell within the WHO permissible limit (< 10 NTU) for surface waters (WHO, 2017), indicative of uncompromised water quality based on turbidity level. The pH levels values were not significantly different across the study sites and were within the WHO maximum permissible limits (6.5–9) for the health ecosystem (WHO 2017). Freshwater lakes with a pH > 7 are alkaline and are dependent on the formation and nature of soils close to the water source or bedrock [27].

The NO_3_-N concentration values didn’t exceed 1 mg/L; the WHO recommended NO_3_-N limits for drinking water (WHO 2017). These results may be attributed to the organic matter decomposition, activities of nitrogen-fixing bacteria in the Lake and surface runoff during heavy rainfall events. Similarly, Wang et al. (2020) reported high NO_3_-N concentration discharge into the DZ River in Houzhai catchment during the early rainfall events, which confirms the assertion that NO_3_-N level in lakes, tend to increase in the rainy season than the dry season. Variations in SRP values are attributed to the recent excessive use of fertilizers rich in phosphorus. Likewise, the recorded SRP values in the current study were slightly higher than values recorded by Tibihika et al. [27] at Lake Bunyonyi.

### Spatial variations of *E. coli* and enterococci concentration

Significant spatial variation of *E. coli* concentration recorded during the study period indicates the heterogeneous distribution of faecal contamination. *E. coli* and enterococci counts at the study stations close to Mugyera and Harutinda Trading Centres were relatively high. This is possibly attributed to the rural people whose pit latrines are constructed less than 20 m from the lake, the possible contamination from the hotels constructed on the Lake peripheral (e.g. Crater Bay Cottage and Lake Bunyonyi Overland Resort) and the direct deposition of human faecal matter from swimmers since the Station near Harutinda Trading Centre is popular for swimming. The detection of *E. coli* and enterococci in water samples indicates faecal contamination by people and warm-blooded animals at various points. Although the levels of *E. coli* and enterococci were not higher than WHO recommended levels for swimming (33 enterococci and 200 *E. coli*), their presence indicate the possibility of occurrence of pathogenic bacteria that cause diseases such as gastroenteritis, cholera, dysentery and typhoid fever after ingestion of contaminated water [10]. Similar to our study results, previous studies attributed high FIB concentrations in the water close to sources from animals and sewage leakages, stormwater runoff, sewage overflows, application of manure in the water catchment area [10, 25, 34]. The effect of pit latrines on the quality of water was also reported by Islam et al. [13] in the Ganges Atrai floodplains of Bangladesh. It was found out that pit latrines enhanced bacterial contamination of adjacent shallow tube well water and that hydrogeological conditions played important role in the transport of FIB bacteria into the water [13].

### Temporal variations of *E. coli* and enterococci concentration

The *E. coli* and enterococci counts were high during the wet season than the dry season. The observed high *E. coli* and enterococci counts in the wet season are linked to runoffs that carry fresh animal manure and human faecal matter. For instance, during heavy rainfall events, the surface runoff from campsites and business centres around the Lake introduce faecal contamination into surface waters. Besides, the stormwater in the Lake catchment sometimes overloads the capacity of toilet facilities and sewage systems, resulting in the unintentional leakage of faecal matter into the lake. Between October and December 20,019 before travel restrictions to Uganda due to the COVID-19 pandemic, the number of tourists visiting Lake Bunyonyi was at its highest peak and the Lake was fully utilized for recreation. Based on our one-year observations, it can be stated that the fluctuations in the number of tourists visiting Lake Bunyonyi are considered as one of the factors affecting the seasonal variation of FIB counts in the Lake. The results corroborate with many other findings on freshwater lakes; for example, Abia et al. [1], Aragonés et al. [4], Chávez-Díaz et al. [6], Edokpayi et al. [8] who reported higher FIB concentrations during the wet season than in the dry season. Similarly, Islam et al. [12] revealed a clear seasonality in FIB concentrations with higher mean concentrations occurring during the wet season (July to October) of 2014–2015. Nguyen et al. [24] attributed seasonal variations in FIB concentration to differences in land use activities, availability of nutrients, and human population growth. Similarly, Kayembe et al. [15] attributed high FIB concentration in water sources during the wet season to the higher runoff and overflow of onsite sanitation systems such as pit latrines and septic tanks into the water sources.

### Comparison of FIB concentration with water quality standards

Results indicated that the Lake understudy was contaminated with faecal matter, whose FIB concentrations fell below the USEPA [30] threshold limit for recreational waters. According to the USEPA [30] directives, the safety threshold for *E. coli* concentration for fresh waters recreational activities was at a geometric mean of 126 CFU/100 mL in multiple samples and 235 CFU/100 mL in single samples. For enterococci concentrations, the safety threshold is at a geometric mean of 33 CFU/100 mL in multiple samples in a 30-day interval and 104 CFU/100 mL in single samples [30]. Our results, therefore, show that Lake Bunyonyi meets standards for recreational activities. Nevertheless, the use of Lake Bunyonyi for recreational purposes is recommendable since the FIB fell under the USEPA [30] threshold for recreational freshwater waters. On the other hand, the Lake’s FIB counts exceeded the threshold limits for drinking water as per the EU Directive 2020/2184, WHO [32], APHA [3], and UNBS [29]. These guidelines require water for human consumption/drinking be free from *E.coli* and enterococci i.e. 0 CFU/100 mL [3, 5, 29, 32]. Similar to results, frequent violation of drinking water quality standards has been reported in previous studies [12, 23]. These results suggest treatment of water derived from Lake Bunyonyi to improve its safety for use. This is because of faecal contamination and possibly, pathogens that may cause waterborne disease outbreaks are present.

The Spearman rank correlation revealed a significant positive relationship between *E. coli* and enterococci in Lake Bunyonyi. These results were expected and indicate the effect of anthropogenic pressure in form of human and animal faecal matter inflow. Similarly, Lenart-Boroń et al. [19] associated FIB variability with surface runoff and supply of ions and bacteria emanating from snowmelt water. Similar results of significant positive correlations between *E. coli* and enterococci bacteria were already reported in other studies by Xue et al. [33], Jeon et al. [14], Tiémoko et al. [28] and Edokpayi et al. [8]. The significant positive correlation of FIB with DO and turbidity is possible because due to the mixing effect of the lake currents. Freshwater bodies with high DO and turbidity favor the survival of FIB concentrations for a relatively long period.

## Conclusion

Based on the study results, we conclude that the observed *E. coli* and enterococci counts in the samples collected from Lake Bunyonyi render them bacteriologically unsuitable for drinking unless treated since they can pose health risks to consumers. The poorly constructed pit latrines in the lake’s watershed and surface runoff following heavy rainfall events are the major sources of faecal pollution in the studied stations. Since 97.1% of analyzed water samples fell below the USEPA permissible intended level or water intended for recreational purposes, this water can be used for recreational purposes and other domestic purposes like washing. The detection of FIB far below the USEPA thresholds for bathing/swimming is enough to recommend the use of Lake Bunyonyi for recreational activities. The measured FIB concentrations were significantly high in the wet season implying that rainfall in the lake catchment increased FIB concentration in the lake water. The detected high levels of FIB at Mugyera and Harutinda stations ( between October and December 2019) are adequate to lure decision-makers and researchers to take strict measure to stop further deterioration, and conduct more research, respectively to ensure sustainability in the utilization of the Lake and safeguard the populace from the possible outbreak of water bone diseases. Additionally, creating local awareness is vital for the effective management of pollution and its health-associated risks such as the danger of swimming in faecal contaminated waters. Additionally, the detected levels of FIB across the study stations are adequate to lure decision-makers and researchers to take strict measures to stop further deterioration and conduct more research, respectively to ensure sustainability in the utilization of Lake Bunyonyi.

## Data Availability

All data generated and analyzed during this study are included in this published article.

## List of Abbreviations

WHO: World Health Organization
APHA: American Public Health Association
FIB: Faecal indicator bacteria
CFU: Colony-forming unit
NTU: Nephelometric turbidity unit
USEPA: The United States Environment Protection Agency
